# Increased Dynamic Amplitude of Low Frequency Fluctuation in Primary Insomnia

**DOI:** 10.3389/fneur.2020.00609

**Published:** 2020-06-30

**Authors:** Xianyun Meng, Jianjun Zheng, Yingpeng Liu, Yi Yin, Kelei Hua, Shishun Fu, Yunfan Wu, Guihua Jiang

**Affiliations:** ^1^Hwa Mei Hospital, University of Chinese Academy of Sciences, Ningbo, China; ^2^Ningbo Institute of Life and Health Industry, University of Chinese Academy of Sciences, Ningbo, China; ^3^Department of Medical Imaging, Guangdong Second Provincial General Hospital, Guangzhou, China

**Keywords:** primary insomnia, functional magnetic resonance imaging, intrinsic brain activity, temporal variability, dynamic amplitude of low frequency fluctuation

## Abstract

The physiological mechanism underlying primary insomnia (PI) is poorly understood. Resting-state functional magnetic resonance imaging (fMRI) has emerged as a powerful tool to explore PI. However, previous studies ignore the dynamics of the brain activity. In the current study, we aimed to explore altered dynamic intrinsic brain activity in PI. Fifty-nine patients with PI and 47 matched healthy controls (HCs) were recruited and underwent resting-state fMRI. The variance of dynamic amplitude of low frequency fluctuation (dALFF) maps across time was calculated to measure the temporal variability of intrinsic brain activity and then compared between patients with PI and HCs. As a result, patients with PI presented increased variance of dALFF in the bilateral hippocampus extending to the parahippocampus, the right putamen and the right anterior insula cortex. In addition, the variance of dALFF in the right putamen was positively correlated with Self-rating Anxiety Scale (SAS) score in PI. Our results revealed increased instability of intrinsic activity in PI.

## Introduction

As the most common sleep disorder, primary insomnia (PI) affects ~30–35% people all around the world (1). The typical characteristic of PI is difficulty falling asleep and feelings of non-restorative sleep (2), this situation usually lasts more than 1 month (3). Associated symptoms with PI such as mood disruption, daytime fatigue and cognitive impairments seriously affect subjects' quality of life (4). However, the physiological mechanism underlying PI is poorly understood.

The pathophysiology of PI has consistently been hypothesized to be related to the hyperarousal in insomniacs (5–8). In recent years, resting-state functional magnetic resonance imaging (fMRI) emerges as a powerful tool to explore the neuromechanism of PI (9). As a novel non-invasive imaging technique for measuring spontaneous brain activity (10), fMRI has been used to find new evidences supporting the hyperarousal hypothesis (11). For example, Chen et al. found greater involvement of anterior insula cortex (AIC) with salience network as well as insula-associated high-frequency gamma power during rest in patients with insomnia (12). Under normal conditions, regions such as the posterior cingulate cortex, the parahippocampal gyrus, and the medial prefrontal cortex in the default mode network (DMN) decrease their activity from wakefulness to slow wave sleep (13). However, activity in the DMN is found altered in sleep deprivation in non-clinical populations (14). The arousal in the DMN is significantly higher in patients with PI even during the day (15, 16). Although these studies deepen our understanding of PI, they ignore the dynamics of the brain activity.

An increasing number of evidences prove that activity and function of the brain is dynamic even during resting state (17). Many methods are proposed to explore the dynamics of the brain (18, 19). One newly developed method named dynamic ALFF (dALFF) is designed to quantify the temporal variability of amplitude of intrinsic brain activity (18). Different from traditional ALFF assuming that brain activity is stationarity, dALFF pays attention on the temporal information of the brain (20). The dALFF depends on simultaneous EEG power fluctuations and can be modulated by non-invasive brain stimulation (21). Exploring the dynamics of the brain activity provides distinct insight into the physiological mechanism of the brain (19, 22, 23). For example, Liao et al. find that combining static and dynamic functional connectomics can differentiate between patients with depression with and without suicidal ideation (24). To our knowledge, few studies have explored altered dynamic intrinsic brain activity in PI.

In the current study, we aimed to explore altered dynamic intrinsic brain activity in PI. Fifty-nine patients with PI and 47 healthy controls (HCs) were recruited and underwent resting-state fMRI. Dynamic ALFF maps of each subject were calculated then the variance of these maps across time was calculated to measure the temporal variability of intrinsic brain activity. In line with previous findings, regions in the DMN and the SN which are consistently found altered might also present altered dynamic intrinsic brain activity in PI.

## Materials and Methods

### Participants

Patients with PI were recruited from the Department of Neurology at Guangdong Second Provincial General Hospital, Guangzhou, China. Patients were diagnosed according to Diagnostic and Statistical Manual of Mental Disorders, Fourth Edition (DSM-IV) for diagnosis of PI. The inclusion criteria for patients were (a) patients had been complaining of difficulty falling asleep, maintaining sleep, or early awakening for at least 1 month; (b) patients had no other sleep disorders such as hypersomnia, parasomnia, sleep related movement disorder or other psychiatric disorders; (c) patients were younger than 60 years old; (d) free of any psychoactive medication at least 2 weeks prior to and during the study; (e) women who were not pregnant, nursing, or menstruating; (f) the insomnia disorder was not caused by organic disease or severe mental disease such as secondary to depression or generalized anxiety.

Healthy controls (HCs) were recruited from the local community by using advertisements. HCs must met the following criterion: (a) Insomnia Severity Index(ISI) score was <7; (b) no history of swing shifts, shift work, or sleep complaints; (c) no medication or substance abuse (including caffeine, nicotine and alcohol); (d) no brain lesions or prior substantial head trauma; (e) no history of phychiatric or neurological diseases.

Finally, 59 patients with PI and 47 HCs (matched for age, gender, and education) were included in the current study. The Insomnia Severity Index(ISI) (25), Pittsburgh Sleep Quality Index (PSQI) (25, 26) and Epworth Sleepiness Scale (ESS) (27) were used to measure the sleep quality of subjects. The Self-rating Anxiety Scale (SAS) (28) and the Self-rating Depression Scale (SDS) (29) scores were also gotten from each subject. All scales used in the current study were validated Mandarin version.

The current study was approved by the ethics committee of the Guangdong Second Provincial General Hospital. Before the study, written informed consents were obtained from all subjects.

### Data Preprocessing

Functional images were preprocessed using Data Processing Assistant for Resting-State fMRI package (DPARSFA, http://www.restfmri.net). The first 10 volumes were removed and followed by slice timing and realignment. The mean frame-wise displacement (FD) was calculated for each subject (30, 31). Subjects were excluded if the translational and rotational displacement exceeded 3.0 mm or 3.0°. No subjects were excluded in this step. The functional images were normalized to the standard EPI template (resampled into 3 × 3 × 3 mm^3^) and smoothed with 6 × 6 × 6 mm^3^ full width at half maximum (FWHM) Gaussian kernel. Detrended to reduce low-frequency drift. Then nuisance covariates including Friston 24 motion parameters (32), white matter signal and cerebrospinal fluid signal were regressed out. Then temporal band-pass filter (0.01–0.1 Hz) was performed. Finally, to remove the effect of head motion and ensure the contiguous time points, scrubbing with cubic spline interpolation was used. We also calculated the mean frame-wise displacement (FD) for each subject (30). There was no significant difference of mean FD between patients with PI and HCs (*p* = 0.161 for two sample *t*-test).

### Calculation of Dynamic ALFF

**Figure 1:**
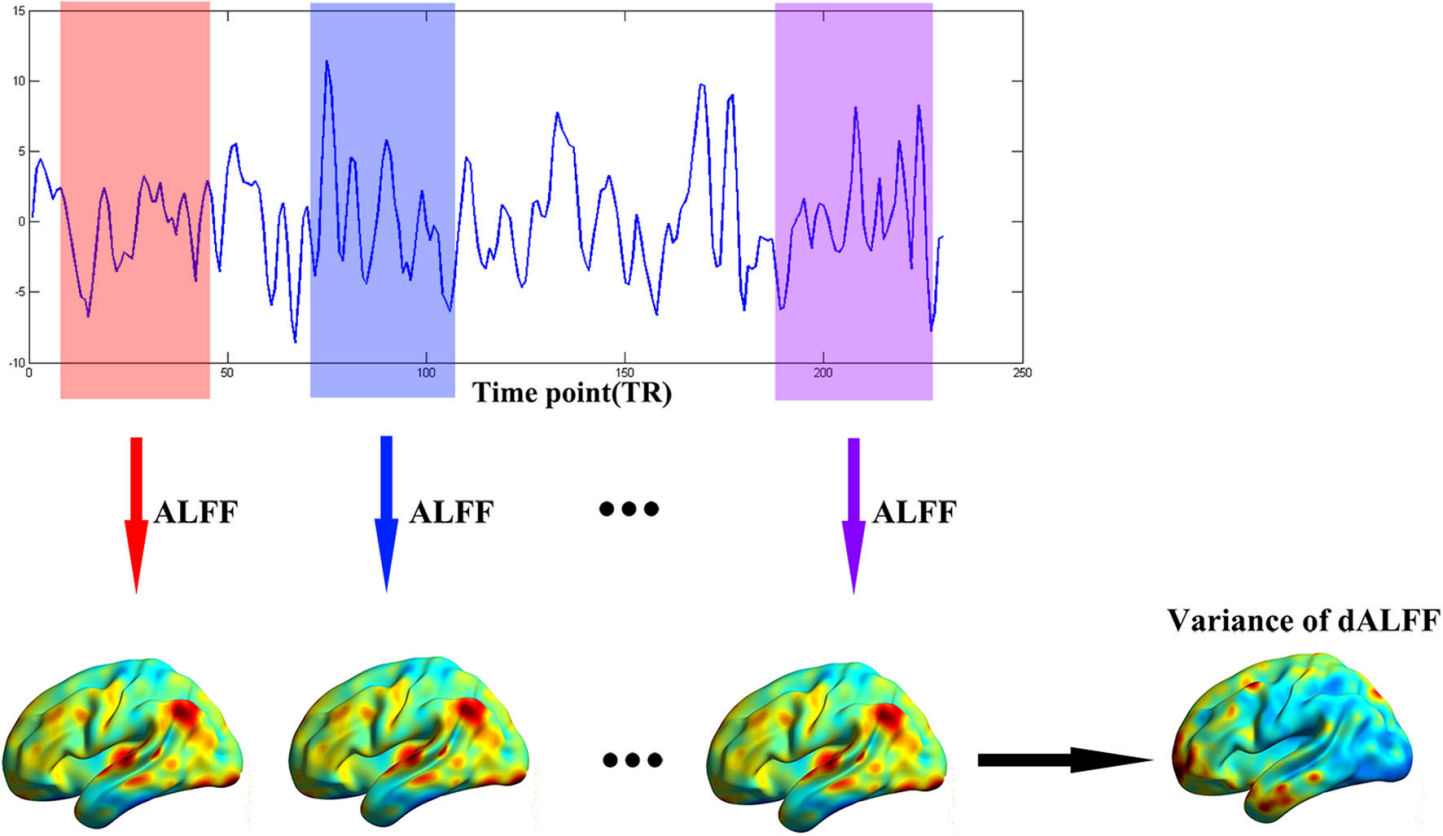
The flow chart of our method. The full-length BOLD fMRI series was segmented into numbers of sliding windows which was represented in different colors. For each sliding window, the ALFF was calculated for each voxel. Last, the variance of dALFF maps across the sliding windows was computed.

The dynamic ALFF was calculated with a sliding window method equipped in DynamicBC (v1.1, www.restfmri.net/forum/DynamicBC) (33). The window length was set to 22 TRs (44 s) with 0.6 overlap (34, 35). The ALFF map was calculated divided by the global mean ALFF value for each window. Thus, we got a series dynamic ALFF maps for each subject. Finally, the variance of dALFF maps across time was calculated to measure the temporal variability of intrinsic brain activity (Figure 1). To exclude effect of parameters selection, we validated our results with different window length (50 TRs, 0.6 overlap).

### Statistical Analysis

Two sample *t*-test was conducted to determine altered dynamic ALFF in patients with PI compared with HCs. In this procedure, age, sex, and years of education were treated as nuisance covariates. Results were corrected with the Gaussian random field (GRF) where threshold of voxel-wise *p* < 0.005 and cluster-level *p* < 0.05.

### Relationship With Symptom Severity

To investigate the correlation between the altered dALFF and clinical symptoms, linear regression between the altered dALFF and clinical symptoms scores (including ISI, PSQI, SAS, SDS) was built. The altered dALFF was defined as the mean values of peak coordinates with a spherical radius of 6 mm (the MNI coordinates were shown in Table S1).

### Validation

To exclude the effect of head motion on our results, we calculated the correlation between altered dynamic ALFF and mean FD in patients with PI.

We also validated our results with different parameters selection (window length = 50 TRs, 0.6 overlap).

## Results

### Demographic and Clinical Characteristics

There was no significant difference between patients with PI and HCs in terms of sociodemographic characteristics, such as age, gender, and year of education. Most of HCs presented normal SAS and SDS scores (SAS or SDS scores <50), except four (or five) had mild anxiety (or depression). Patients with PI presented higher ISI, PSQI, SAS, and SDS scores than those of HCs (all *p* < 0.001) (Table 1).

**Table 1:** Sociodemographic characteristics of patients with PI and HCs.

	**PI (*n =* 59)**	**HC (*n =* 47)**	***p***
Age (years), mean ± SD	39.27 ± 10.72	40.02 ± 9.15	0.704^a^
Gender, male: female	26:33	14:33	0.192^b^
Years of education, mean ± SD	7.47 ± 3.58	8.30 ± 4.21	0.279^a^
Handedness, right/left	59/0	47/0	–
PSQI	12.41 ± 3.31	5.83 ± 2.27	<0.001^a^
SAS	51.83 ± 10.78	42.72 ± 5.78	<0.001^a^
SDS	55.37 ± 8.90	44.04 ± 6.48	<0.001^a^
ISI	19.67 ± 3.32	5.68 ± 2.45	<0.001^a^
ESS	8.55 ± 6.38	9.02 ± 5.03	0.678^a^

a two-tailed two sample t-test;

b *Chi-square t-test; HC, healthy control; PI, primary insomnia; PSQI, Pittsburgh Sleep Quality Index; SAS, Self-rating Anxiety Scale; SDS, Self-rating Depression Scale; ISI, Insomnia Severity Index; ESS, Epworth Sleepiness Scale*.

### Increased dALFF in Patients With PI

**Figure 2:**
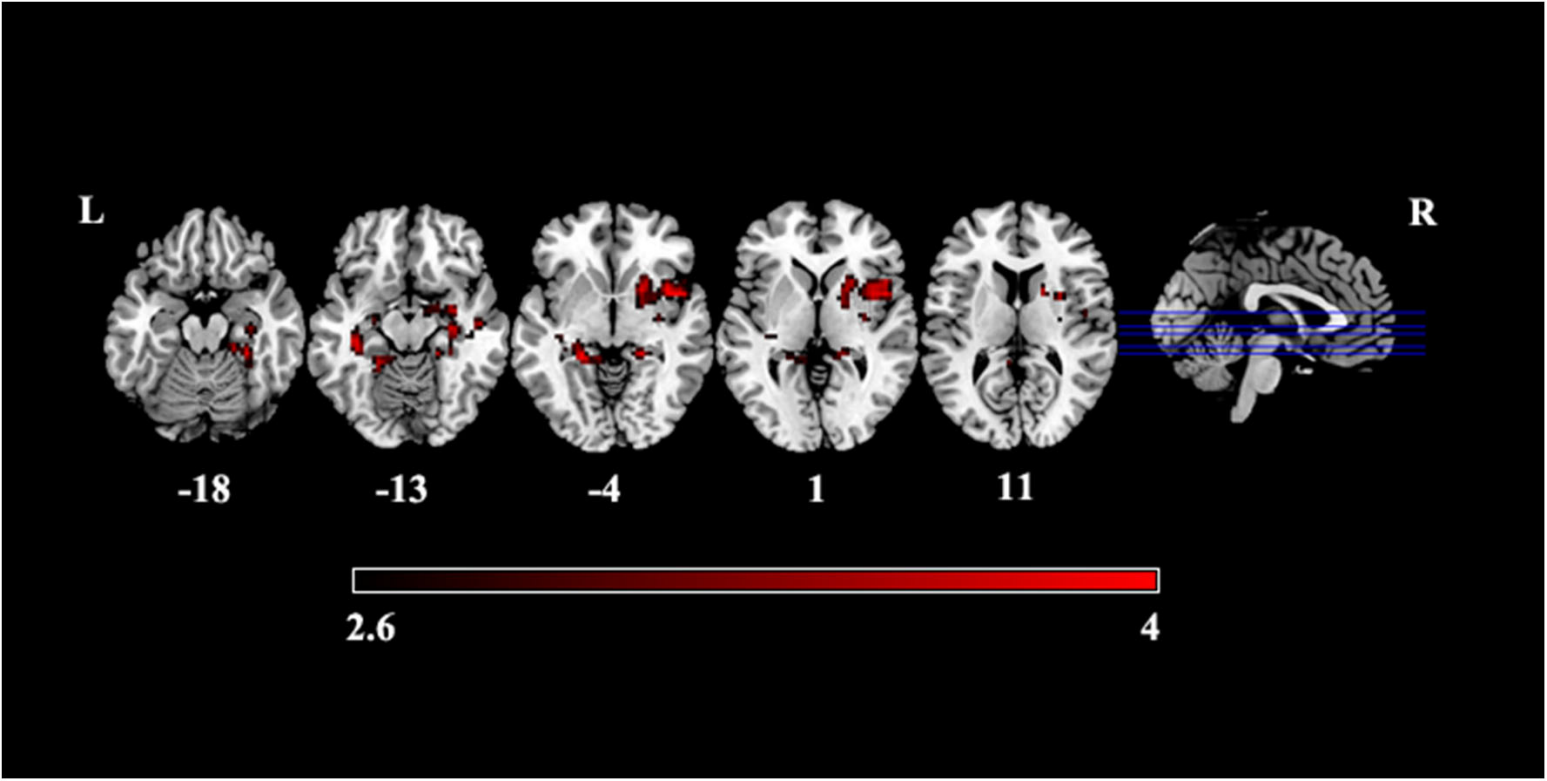
Increased variance of dALFF in patients with PI.

Compared with HCs, patients with PI presented significantly increased variance of dALFF in subcortical regions including the bilateral hippocampus extending to the parahippocampus, the right putamen and the right anterior insula cortex (Figure 2, Table 2).

**Table 2:** Significant differences in dALFF between patients with PI and HCs.

	**Clutsers**	**Voxels**	**Regions**	**MNI (x, y, z)**	**T**
PI > HC	1	216	L.Para	−21, −39, −3	4.74
			L.Hippocampus		
	2	357	R.AIC	29, 3, 3	4.08
			R.Putamen		
			R.Hippocampus		

*The MNIs were the peak coordinate in clusters*.

*L.Para, left parahippocampus; R,AIC, right anterior insula cortex*.

### The Increased Variance of dALFF Was Correlated With SAS Score

**Figure 3:**
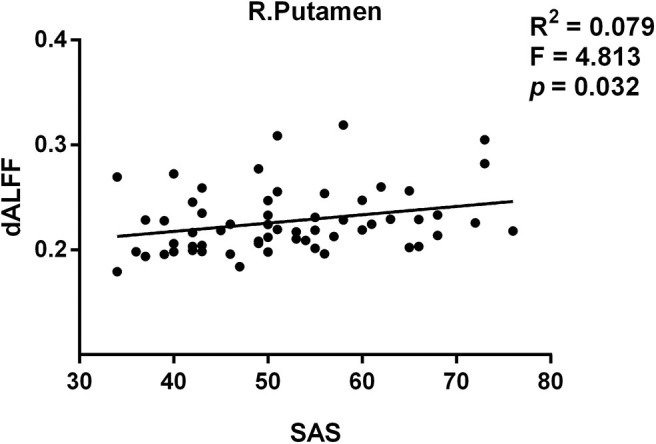
The relationship between the variance of dALFF and SAS score in PI.

Moreover, we found the variance of dALFF values were associated with SAS score in patients with PI. The peak coordinates of the increased dALFF was in Table S1. The variance of dALFF of the right putamen (*R*^2^ = 0.079, *p* = 0.032 uncorrected, *F* = 4.813) was significantly associated with SAS score (Figure 3).

### Validation Results

To exclude the effect of head motion, we also calculated the correlation between the altered variance of dALFF and mean FD. There was no significant correlation between the variance of dALFF and mean FD (all *p* > 0.05). In addition, there was no significant difference of mean FD between patients with PI and HCs (*p* = 0.161).

Our main results could be validated with different parameter selection (the details were shown in Table S1).

## Discussion

In the current study, it was the first time to explore altered dynamic intrinsic brain activity in PI using dALFF. Patients with PI presented increased variance of dALFF in the bilateral hippocampus extending to the parahippocampus, the right putamen and the right anterior insula cortex. In addition, the variance of dALFF in the right putamen was correlated with SAS score in PI. In line with the hyperarousal hypothesis, we found that regions in the DMN presented increased variance of dALFF in patients with PI. Our results revealed increased instability of intrinsic activity in PI.

Dynamic intrinsic brain activity of regions in the DMN was more unstable in patients with PI compared with HCs. Previous studies consistently found structural and functional aberrance in the DMN. For example, as a key region of the DMN, structural and functional aberrance of the bilateral hippocampus was consistently found (11). Evidences from animals (36) and human (37) supported that this aberrance was resulted from sleep restriction or deprivation (36, 37). The attenuated neurogenesis in the hippocampus resulted in a deterioration of neurocognitive performance by disrupting the hippocampus-dependent memory consolidation in PI (38), even in normal sleepers with prolonged sleep deprivation (39) and chronic insomnia (40). In addition to structural aberrance in the hippocampus, altered activity in the DMN including the hippocampus is also found in sleep deprivation (14). The sleep-wake differences in cerebral metabolic rate for glucose of the hippocampus in PI is smaller than that in HCs (41). Functional connectivity of the hippocampus connected to the middle frontal gyrus was found altered and this altered link was correlated with insomnia severity (42). Decreased functional connectivity of the parahippocampal gyrus connected to the posterior DMN node was also found in PI (13). However, few studies focused on altered dynamic intrinsic brain activity in PI. In addition to these findings, we found the temporal intrinsic brain activity of the hippocampus and the parahippocampal gyrus was more unstable in patients with PI compared with HCs. Consist with previous studies, the increased variance of dALFF might be related with rumination in PI (15).

In addition, the increased variance of dALFF of the right AIC was also found in patients with PI. The AIC was posited to be a source of the slow waves that characterized deeper stages of sleep (43). Its involvement with the SN was consistently found greater in patients with PI (12) and in insomnia in rodents (44). The correlation between the AIC BOLD and EEG gamma frequency power was also greater reflecting negative affect in PI (12). While, the effective connectivity of the AIC was found decreased in PI (45). Combing with these evidences, the increased variance of dALFF might reflect overactive but ineffective activity of the AIC in PI.

We also excluded the effect of head motion and parameter selection on our results. There was no significant correlation between mean FD and altered dALFF in PI and no significant difference of the mean FD between PI and HCs. Yan et al. (46) also did not find any meaningful relationships between dALFF with physiological signals. All these results suggested the head motion did not contribute observed results in the current study to some extent. In addition, we validated our results with different parameters selection suggesting the increased dALFF in patients in PI was reliable.

There were many limitations must be considered. First, results obtained come from a single dataset with limited sample size. Future study could use another independent sample set to validate our results. Second, even though abnormal emotional function was a predisposing factor to PI but not the consequence of sleep less (47, 48), we could not exclude the possibility that the increased dALFF found in our results was more relative to the abnormal emotional function in PI. More work had to be done to elaborate the role of altered dALFF in the neuromechanism of PI. Finally, lacking of polysomnography of patients with PI was another weakness.

## Conclusion

In the current study, we explored altered dynamic intrinsic brain activity in PI using dALFF. We found that patients with PI presented increased variance of dALFF in the bilateral hippocampus extending to the parahippocampus, the right putamen and the right anterior insula cortex. In addition, the increased variance of dALFF values in the right putamen was correlated with SAS score in PI. Our results revealed increased instability of intrinsic activity in PI.

## Data Availability Statement

The datasets generated for this study are available on request to the corresponding author.

## Ethics Statement

The studies involving human participants were reviewed and approved by The ethics committee of Guangdong Second Provincial General Hospital. The patients/participants provided their written informed consent to participate in the research.

## Author Contributions

XM, JZ, and YW: mainly write the paper. YL and YY: are charge of designing the method. KH and SF: are in charge of data acquisition. GJ: design all study. All authors revised the manuscript and final version of manuscript.

## Conflict of Interest

No author has financial or other contractual agreements that might cause conflicts of interest.
