# Supplementary material for: Increased Dynamic Amplitude of Low Frequency Fluctuation in Primary Insomnia
Source: Front Neurol. 2020 Jun 30;11:609. doi: 10.3389/fneur.2020.00609 (PMC7344192; doi:10.3389/fneur.2020.00609)
Supplement: Supplementary file 1 [file Table_1.DOCX]

**Table S1**. We validated our main results (peak coordinates with a spherical radius of 6 mm) with different parameters selection (window length = 50 TRs, 0.6 overlap). Our main results were still significant (all *p* < 0.05).

|  | Regions | MNI | T |
| --- | --- | --- | --- |
| PI > HC | L.Para | -21, -39, -3 | 2.79 |
|  | L.Hippocampus | -36, -30, -9 | 3.05 |
|  | R.AIC | 33, -18, -15 | 2.52 |
|  | R. Putamen | 39, 3, 3 | 2.65 |
|  | R.Hippocampus | 30, 6, 9 | 2.73 |

Note: L.Para, left parahippocampus; R. AIC, right anterior insula cortex.
